# COVID-19 global risk evaluation: rankings, reducing surveillance bias, and infodemic

**DOI:** 10.3389/fpubh.2025.1589461

**Published:** 2025-08-04

**Authors:** Michał P. Michalak, Elżbieta Węglińska, Agnieszka Kulawik, Jack Cordes, Michał Lupa, Andrzej Leśniak

**Affiliations:** ^1^Faculty of Geology, Geophysics and Environmental Protection, AGH University of Krakow, Kraków, Poland; ^2^Faculty of Science and Technology, University of Silesia in Katowice, Katowice, Poland; ^3^Department of Public Health and Community Medicine, Tufts University School of Medicine, Boston, MA, United States; ^4^Faculty of Space Technologies, AGH University of Krakow, Kraków, Poland

**Keywords:** local positivity, global positivity, spearman correlation, ranking, surveillance bias

## Abstract

This study examines how public health institutions estimate regional COVID-19 burdens, pursuing two primary objectives: (1) to analyze the methodologies employed for regional risk assessment, and (2) to perform spatial and Spearman rank correlation analyses of risk metrics that incorporate testing data across 101 countries. Classification methods used to assess COVID-19 risk often treat testing as a secondary, qualitative factor, overlooking its value as a quantitative input. Integrating testing data with case counts can improve the accuracy of regional infection probability estimates. Spatial analysis revealed that probabilistic metrics—such as the local probability of infection—showed stronger spatial synchronization of epidemic patterns compared to observed-to-expected case ratios. The death-to-population ratio displayed the strongest positive correlation with the observed-to-expected cases ratio. Conversely, the case fatality rate exhibited only a weak positive correlation with probabilistic metrics, though these correlations were not consistently statistically significant. The findings underscore the potential of probabilistic metrics, such as the local probability of infection, in predicting COVID-19 risk. Further research is warranted to explore the predictive capacity of probabilistic metrics concerning death-related outcomes.

## 1 Introduction

Regional infectious disease risk rankings play a significant role in the management of the epidemics. The recommendations based on such rankings are intended to minimize the impact of severe disease and prevent strain on healthcare systems by directing priorities on prevention measure implementation (1–3). For example, regions on the top of the risk rankings can be subject to various restrictions including border closures.

We propose two major considerations with respect to the utility of rankings for infectious diseases. Because test results cannot be known in advance, ranking methods should not be sensitive to regional testing intensity. This will prevent punishing countries for implementing large screening strategies and vice versa: countries with low testing rates cannot escape from a fair assessment. The second consideration is that the ranking metric should reflect relative differences in regional health threats to prioritize the implementation of preventive measures. While our study focuses on methodological aspects of ranking robustness—particularly the influence of testing intensity and relative threat levels—we acknowledge that real-world ranking outcomes are also shaped by broader contextual factors, including political decisions (4), healthcare system differences (5), socio-economic conditions (6), and data reporting quality (7).

**Figure 1:**
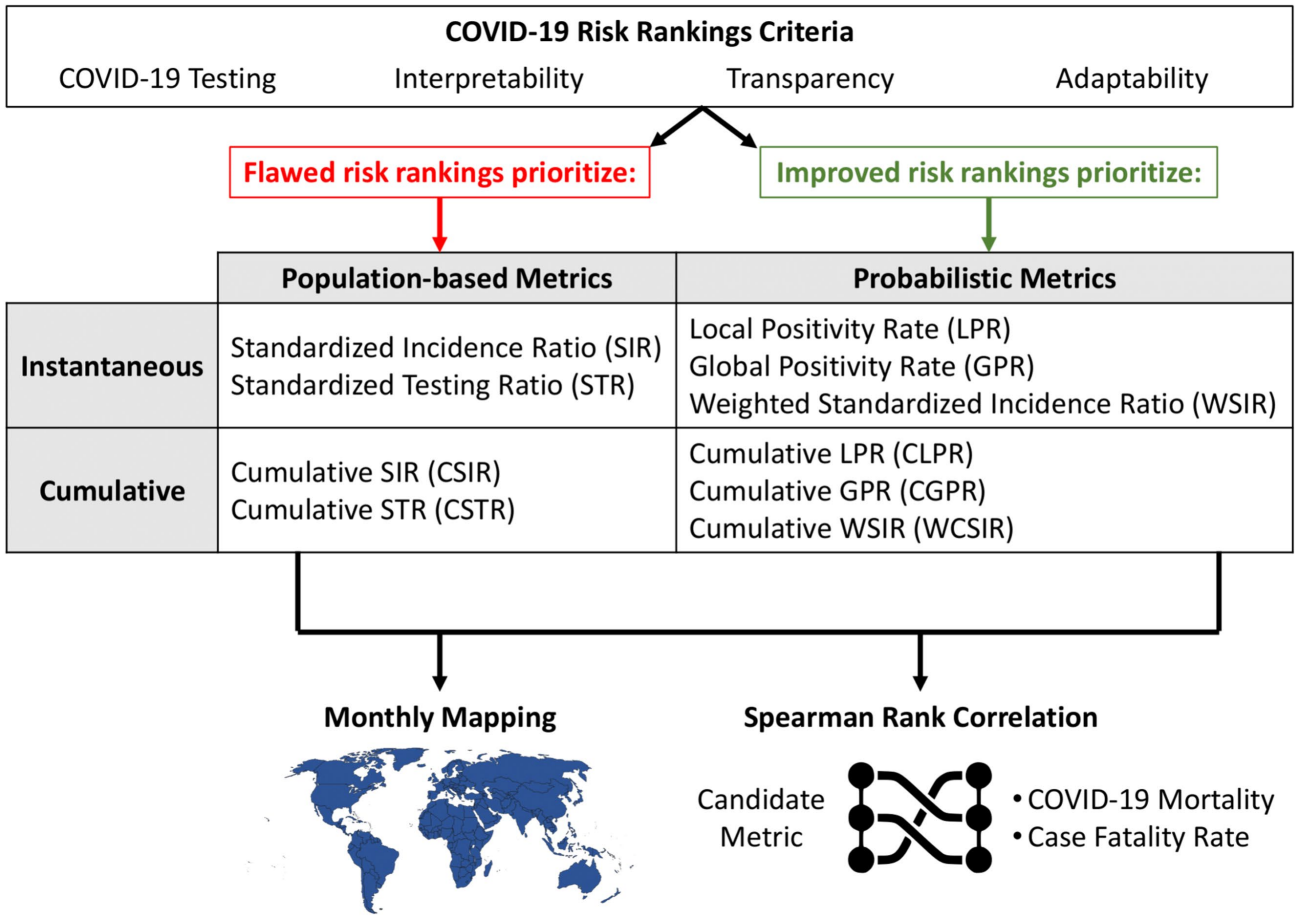
Overview of the study design with three main components. The top section contrasts flawed and improved COVID-19 risk ranking strategies based on their emphasis on either population-based or probabilistic metrics. These metrics are evaluated in two applications: (1) monthly mapping of regional COVID-19 risk and (2) correlation analysis with health outcomes (mortality and case fatality rate) using Spearman rank correlation. The framework supports the identification of interpretable and adaptable candidate metrics for effective public health communication.

In this study, we discuss the advantages and drawbacks of approaches used for COVID-19 risk classifications among public health institutes in terms of the two considerations above as well as transparency and adaptability. We calculated both the ratio of local and global positivity (4) and metrics that do not account for testing intensity across countries and determined their association with countries’ overall COVID-19 death rates and case fatality rates. The summary of the study is presented in Figure 1. A freely available and open-source application (link available at the Computer code availability section) was developed to illustrate the discussed concepts and analyses. We recognize that, while reducing sensitivity to testing differences and focusing on relative health threats are important steps toward fairer rankings, other persistent challenges—such as varying case and death definitions, reporting delays, and differences in healthcare infrastructure—also impact the perceived fairness of global health assessments.

## 2 Risk rankings among public health organizations

In this section, we limit ourselves to a factual description of institutional ranking approaches. Critical evaluation and interpretation are reserved for the Discussion.

### 2.1 The Robert Koch Institute

The Robert Koch Institute is a well-known public health organization that has continually published regional risk classifications during the COVID-19 pandemic (8, 9). In July 2020, the Institute blacklisted Luxembourg due to a large ratio of COVID-19 cases to inhabitants. This blacklisting decision was followed at the time by several European countries including Denmark, Norway, and the Baltic countries (10, 11). The Minister of Foreign Affairs for Luxembourg interpreted Luxembourg’s top position in the risk ranking as a baseless accusation, and argued that infection numbers must be viewed in the context of Luxembourg’s large-scale testing strategy (11). Indeed, while being punished by the Robert Koch Institute, Luxemburg received public praise from the European Centre for Disease Prevention and Control for implementing a large screening strategy which was in addition considered effective in terms of limiting the spread of the virus (12). Ultimately, the decision to blacklist Luxembourg was reverted by the Robert Koch Institute in August 2020 (13).

### 2.2 Centers for Disease Control and Prevention

Another example of flawed risk advisories was the Travel Health Notice (THN) adopted by the United States Centers for Disease Control and Prevention (CDC). It aimed to notify travelers about relative differences in health threats in destinations around the world using a discrete four-point scale (14). The criteria are divided into primary (quantitative) and secondary (qualitative). The quantitative group comprises three measures: (1) case count, (2) incidence rate per 100,000 people, and (3) trajectory (decelerating, slowing or stable). The qualitative group consisted of (1) measures of a destination’s treatment capacity and (2) measures of a destination’s public health infrastructure. Destination risk rankings were based on numeric thresholds for these criteria. For example, Level 3 (High Risk) required the number of cases to be greater than 500 or the incidence rate to be more than 3 per 100,000. Crucially, while qualitative measures did include measures of variables such as testing capacity, actual testing rates were not used in the rankings.

### 2.3 Covid ActNow

Covid ActNow is an initiative of the Act Now Coalition launched in March 2020 with the goal of providing up-to-date COVID-19 risk information for states in the US. From its founding through spring 2022 (15) it used three key metrics: daily new cases, reproductive number, and positive test rate (defined as the percentage of reported COVID-19 PCR testing that was positive). There were also three secondary metrics: intensive-care unit (ICU) capacity used, percent vaccinated, and social vulnerability metrics such as unemployment. We note that this is one of the few approaches that used cases and testing simultaneously in its positivity rate metric; however, it was dropped in spring 2022 (15).

### 2.4 The UK Health Security Agency

The UK Health Security Agency (UKSHA) developed a risk assessment methodology to inform ministerial decisions on red list countries and territories (16). The UKSHA consists of four parts: (1) variant assessment (e.g., transmissibility, severity of disease), (2) triage (e.g., testing rates per 100,000 population, case rates per 100,000 population, positivity rate, strong travel links with countries known to have community transmission of a variant), (3) further risk assessment (criteria assumed as qualitative such as testing strategies, transparency of data, representativeness but also vaccination rates), and (4) outcome–a five-point scale is applied (very low, low, medium, high, very high) to inform the ministerial decisions.

## 3 Methods

We used a publicly available database developed by Emanuele Guidotti recording daily time-series data of COVID-19 cases, deaths, recoveries, tests, vaccinations, and hospitalizations (17). In this paper, we used data for 101 countries aggregated monthly from January 2021 to December 2021. In cases where data was incomplete for a given country during the specified time period, it was excluded from the analysis. The countries included in our analysis account for a total of 53.9% of the global population.

In this study we consider six metrics for each country i summarized in Table 1.

**Table 1:** Classification of risk metrics used in the study.

Metrics	Formulas
Names for local metrics	Formulas for local metrics
Instantaneous local positivity rate	${\mathrm{LPR}}_{i}(t)=\frac{O_{i,t}}{T_{\begin{array}{c} i,t\\ \; \end{array}}}$ , where t denotes taking into account cases confirmed on day t only
Cumulative local positivity rate	${\text{CLPR}}_{i}(t)=\frac{O_{i,1:t}}{T_{\begin{array}{c} i,1:t\\ \;\\ \; \end{array}}}$ , where 1:t denotes summation of cases or tests from the 1st day up to the day t
Names for global metrics	Formulas for global metrics
Instantaneous global positivity rate	$\mathrm{GPR}(t)=\frac{\sum_{i}O_{i,t}}{\sum_{i}T_{i,t}}$
Cumulative global positivity rate	$\text{CGPR}(t)=\frac{\sum_{i}O_{i,1:t}}{\sum_{i}T_{i,1:t}}$
Names for relative metrics	Formulas for relative metrics
Instantaneous standardized incidence ratio	${\mathrm{SIR}}_{i}(t)=\frac{O_{i,t}}{E_{i,t}}$ , where $E_{i,t}=P_{i}*\frac{\sum_{i}O_{i,t}\;}{\sum_{i}P_{i}}$
Instantaneous standardized testing ratio	${\mathrm{STR}}_{i}(t)=\frac{T_{i,t}}{TE_{i,t}}$ , where $TE_{i,t}=P_{i}*\frac{\sum_{i}T_{i,t}\;}{\sum_{i}P_{i}}$
Cumulative standardized incidence ratio	${\text{CSIR}}_{i}(t)=\frac{O_{i,1:t}}{\;E_{i,1:t}}$ , where $E_{i,1:t}=P_{i}*\frac{\sum_{i}O_{i,1:t}\;}{\sum_{i}P_{i}}\;$
Cumulative standardized testing ratio	${\text{CSTR}}_{i}(t)=$ $\frac{T_{i,1:t}}{TE_{i,1:t}}$ , where $TE_{i,t}=P_{i}*\frac{\sum_{i}T_{i,1:t}\;}{\sum_{i}P_{i}}$
Weighted instantaneous standardized incidence ratio	${\text{WSIR}}_{i}(t)=$ $\frac{{\mathrm{SIR}}_{i}(t)}{{\mathrm{STR}}_{i}(t)}=\frac{{\mathrm{LPR}}_{i}(t)}{\mathrm{GPR}(t)}\;$
Weighted cumulative standardized incidence ratio	${\text{WCSIR}}_{i}(t)=$ $\frac{{\text{CSIR}}_{i}(t)}{{\text{CSTR}}_{i}(t)}=$ $\frac{{\text{CLPR}}_{i}(t)}{\text{CGPR}(t)}$

Instantaneous measures use only data reported on the index date, while cumulative measures use the index date data as well as all prior data. For example, the $\mathrm{SI}R_{6}(4)$ uses the observed number of cases for country (e.g., Brazil or Germany) i=6 on day 4 of the pandemic whereas the $\mathrm{CSI}R_{6}(4)$ uses data for days 1 through 4 of the pandemic. The relative measures defined in Table 1 use indirect standardization, the ratio of observed to expected numbers of cases or tests (18, 19). Furthermore, the ${\text{WCSIR}}_{i}$ can be conceptualized as the ratio between local and global positivity (see also Section 5.3).

All four metrics—SIR, STR, WSIR, and WCSIR—use a shared global reference value (a ‘global coefficient’) as part of their construction (Table 1). For SIR and STR, this reference is the global incidence rate and global testing rate, respectively, which are used to compute expected values in each country through indirect standardization. In the case of WSIR and WCSIR, the global positivity rate (or a severity ratio) serves as the benchmark. These measures do not assume spatial homogeneity of infection dynamics across countries, but rather compare local values to a common reference to identify relative deviations. For example, if the global positivity rate is 5%, then a country with 10% positivity would have a WSIR of 2, indicating that its case detection relative to testing is twice as high as the global average. Unlike standardized ratios such as SIR or CSIR, basic positivity measures such as the Local or Global Positivity Rate are directly computed from raw data and do not require a reference value or assumptions about expected incidence.

We used Spearman rank correlation coefficients to determine the association between each metric and two outcome variables: (1) the overall COVID-19 mortality rate and (2) the case fatality rate. The Spearman coefficient compares on countries’ ranking positions for each metric as compared to each outcome. The coefficient ( $r_{s}$ ) is defined in Equation 1.

(1) $$r_{s}=\frac{\mathrm{cov}(R(X),R(Y))}{\sigma_{R(X)}\;\sigma_{R(Y)}}\;$$

where cov(R(X),R(Y)) is the covariance of the rank according to the metric (X) and the rank according to the outcome (Y), and $\sigma_{R(X)},\sigma_{R(Y)}$ are the standard deviations of the rank variables. The absolute value of this coefficient measures the strength of the dependence between considered variables, while the sign points to the direction of the correlation. The threshold for statistical significance was set at an alpha value of 0.05. We performed all computation in R and used the following R packages: dplyr (20) ggplot2 (21), ggpubr (22), plotly (23), gridExtra (24) and readxl (25).

## 4 Results

Due to a large sample size (*n* = 101) and different ranking orders for different metrics, we decided to present a representative sample of countries that captures the most relevant patterns without overwhelming the visual presentation. Countries from each continent were selected to capture the diversity of results across different latitudes, levels of development, and climatic or economic conditions. The selection includes countries with the highest, lowest, and intermediate index values, allowing us to present the full spectrum of the data and to compare both extremes and typical cases. The sample also includes countries of major demographic, economic, or environmental importance (e.g., the USA, India, Germany), which are frequently used as reference points in comparative analyses. Full data are available at our GitHub repository (see Data availability section).

### 4.1 Population-based standardization (SIR and CSIR)

**Figure 2:**
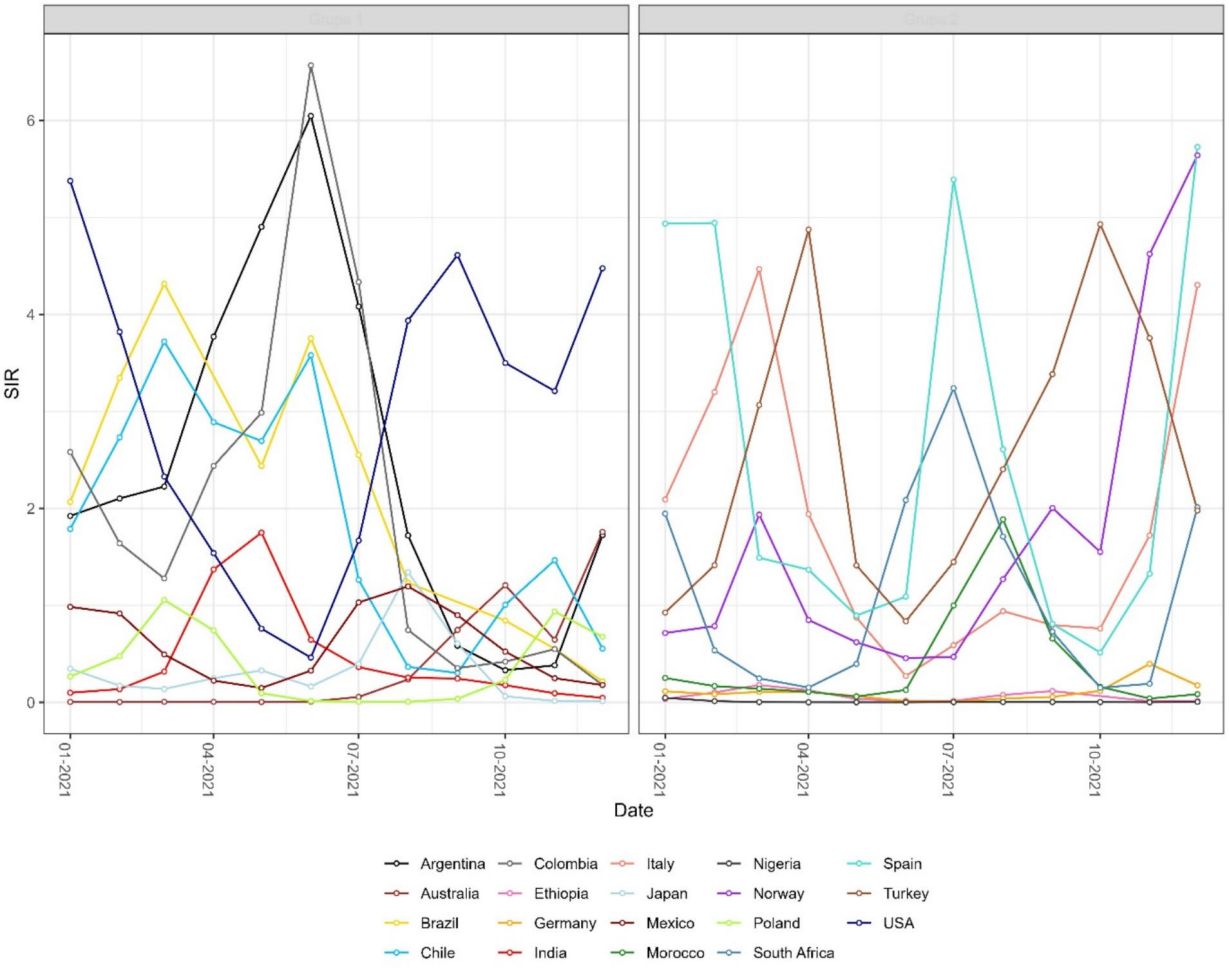
Monthly time series of the instantaneous standardized incidence ratio (SIR) for selected countries, grouped for readability. SIR is defined as the ratio of observed to expected COVID-19 cases. Values above 1 indicate that a country reported more cases than expected.

The highest and lowest SIR values were observed for Peru and Madagascar: 25.09 and 0.0002, respectively (Figure 2). This means that the observed number of cases in Peru was about 25 times greater than the expected number of cases in June 2021. When considering the cumulative number of cases during 2021, the highest ratio of observed to expected number of cases was assessed for Slovakia between January and February 2021 (CSIR = 10.6).

### 4.2 Probabilistic standardization (LPR, WSIR, and WCSIR)

**Figure 3:**
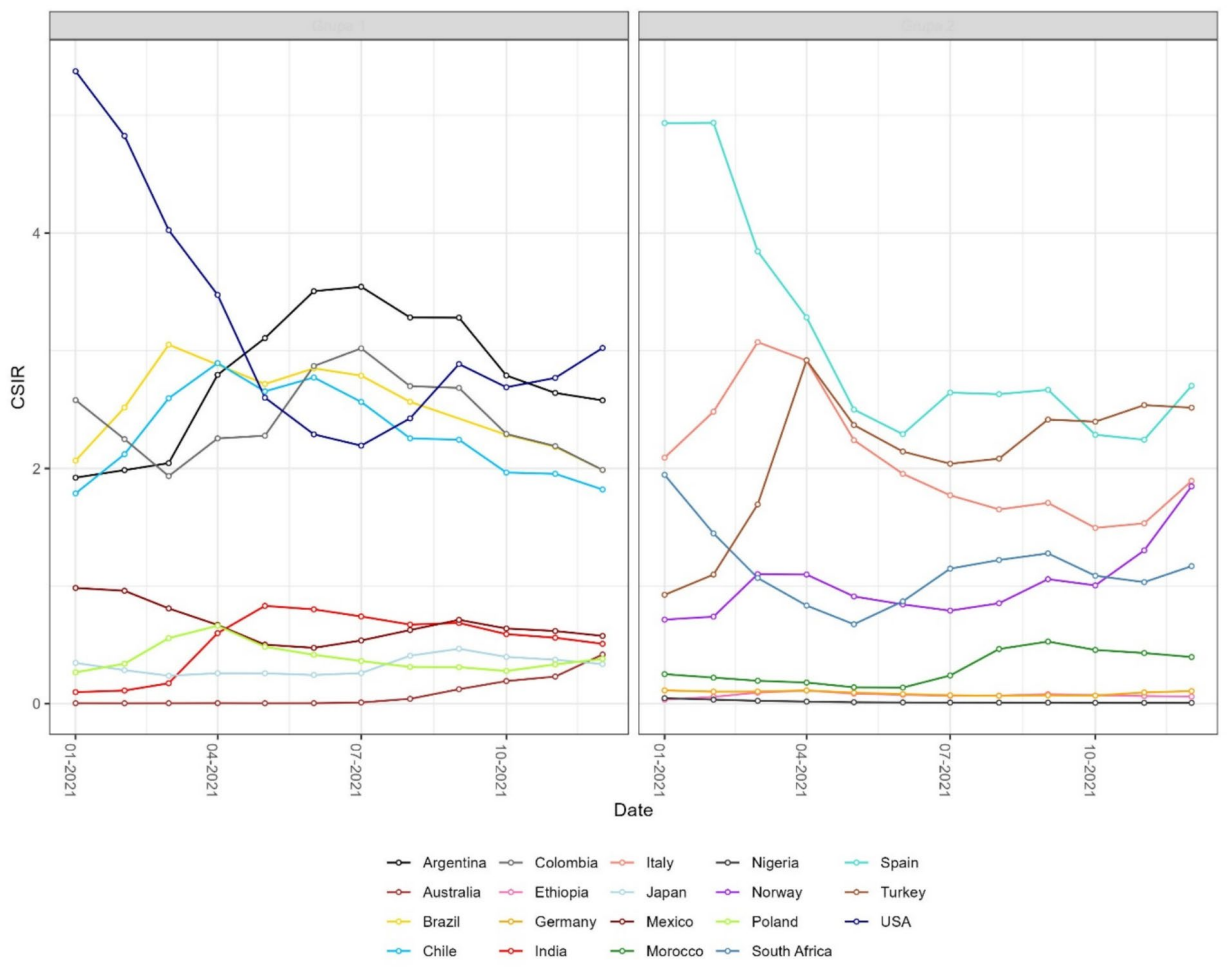
Monthly time series of the cumulative standardized incidence ratio (CSIR) for selected countries. CSIR is defined as the ratio of total observed to total expected COVID-19 cases over time. As a cumulative measure, the resulting curves are smoother than those of the instantaneous SIR shown in Figure 2. Values above 1 indicate that a country reported more cumulative cases than expected.

**Figure 4:**
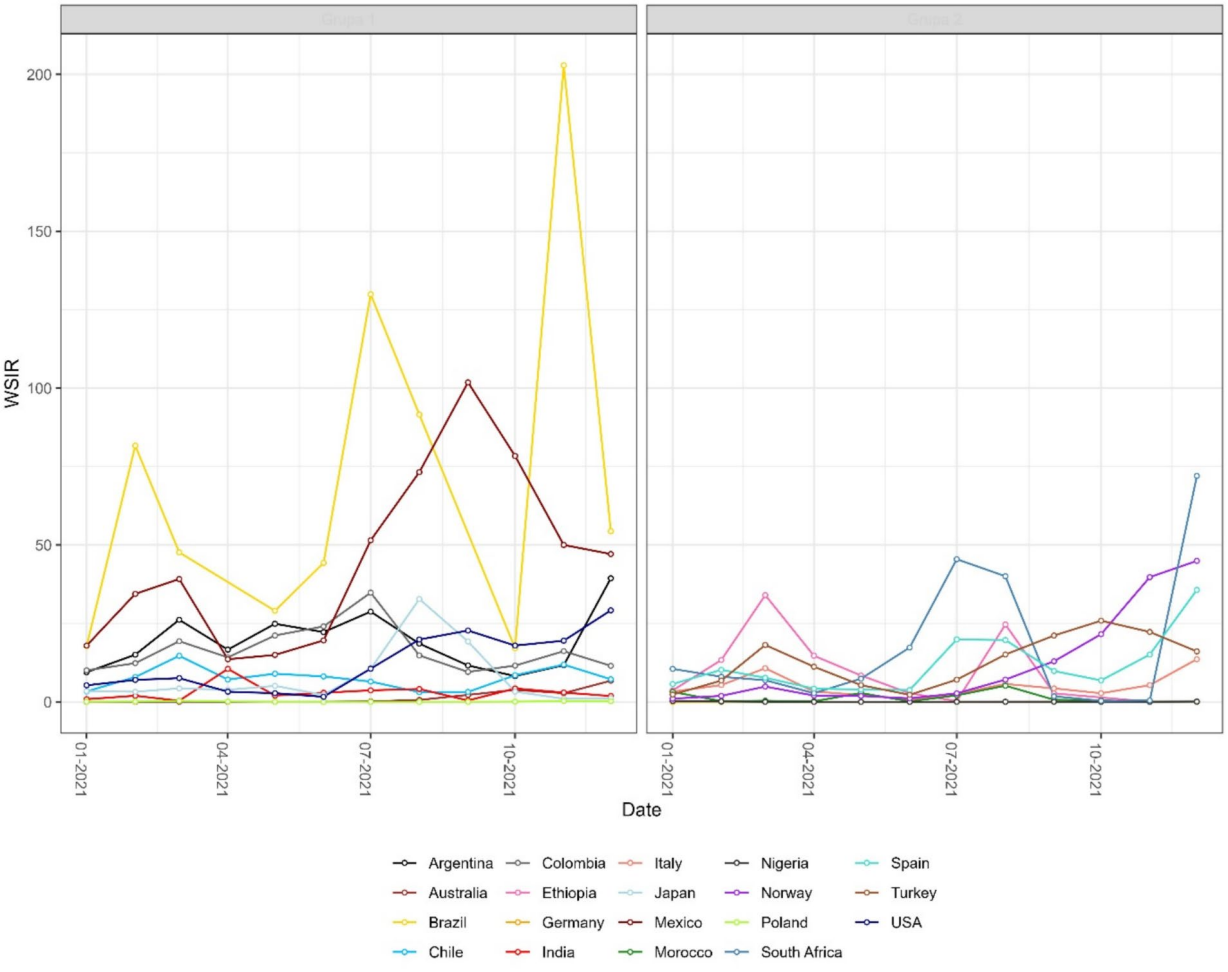
Monthly time series of the weighted standardized incidence ratio (WSIR) for selected countries. WSIR is defined as the ratio of local to global test positivity rates, reflecting the relative detectability of COVID-19 infections across countries. Values above 1 indicate that a country has a higher positivity rate than the global average.

The results observed for the SIR and CSIR (Figure 3) values do not take into account variable testing coverage across countries. The ratio of confirmed cases and conducted tests in the non-cumulative version (WSIR – Figure 4) yielded two countries as the most affected: Brazil (WSIR = 202.87, November 2021) and Guam (WSIR = 196.68, October, 2021). These values can be interpreted two ways: (1) the local positivity (for Brazil) is 202.87 times greater than the global positivity (for all 101 countries), and (2) the relative burden of infection (SIR) is 202.87 times greater than the relative test coverage (STR).

### 4.3 Spatial patterns and phase portrait

**Figure 5:**
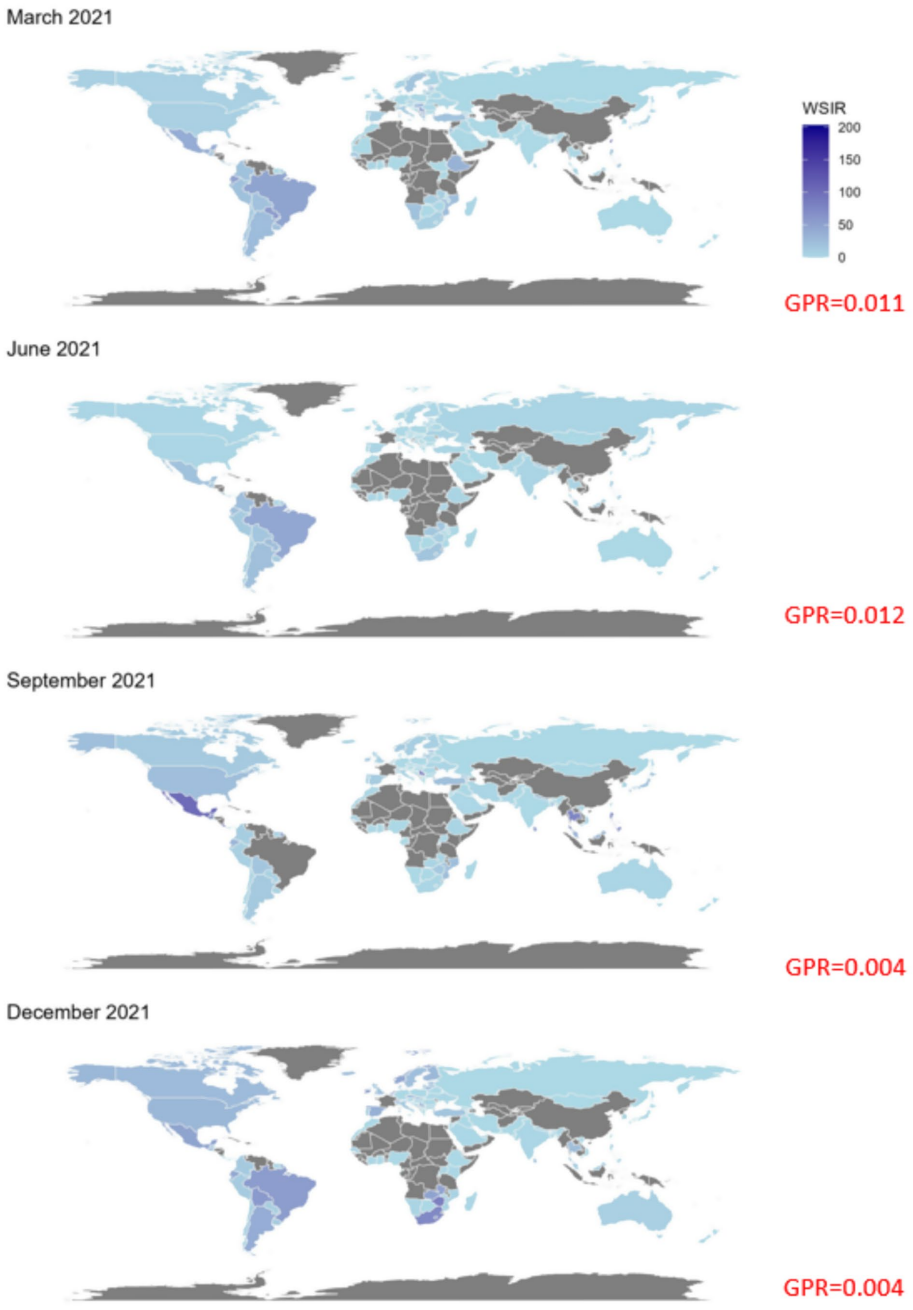
Global maps of the weighted standardized incidence ratio (WSIR) for four selected months in 2021: March, June, September, and December. WSIR is defined as the ratio of local to global COVID-19 test positivity rates, reflecting the relative detectability of infections. Darker shades indicate higher WSIR values, suggesting a combination of under-testing and elevated transmission intensity in those regions. Grey areas represent countries with missing data. The global positivity rate (GPR) for each month is reported in red.

**Figure 6:**
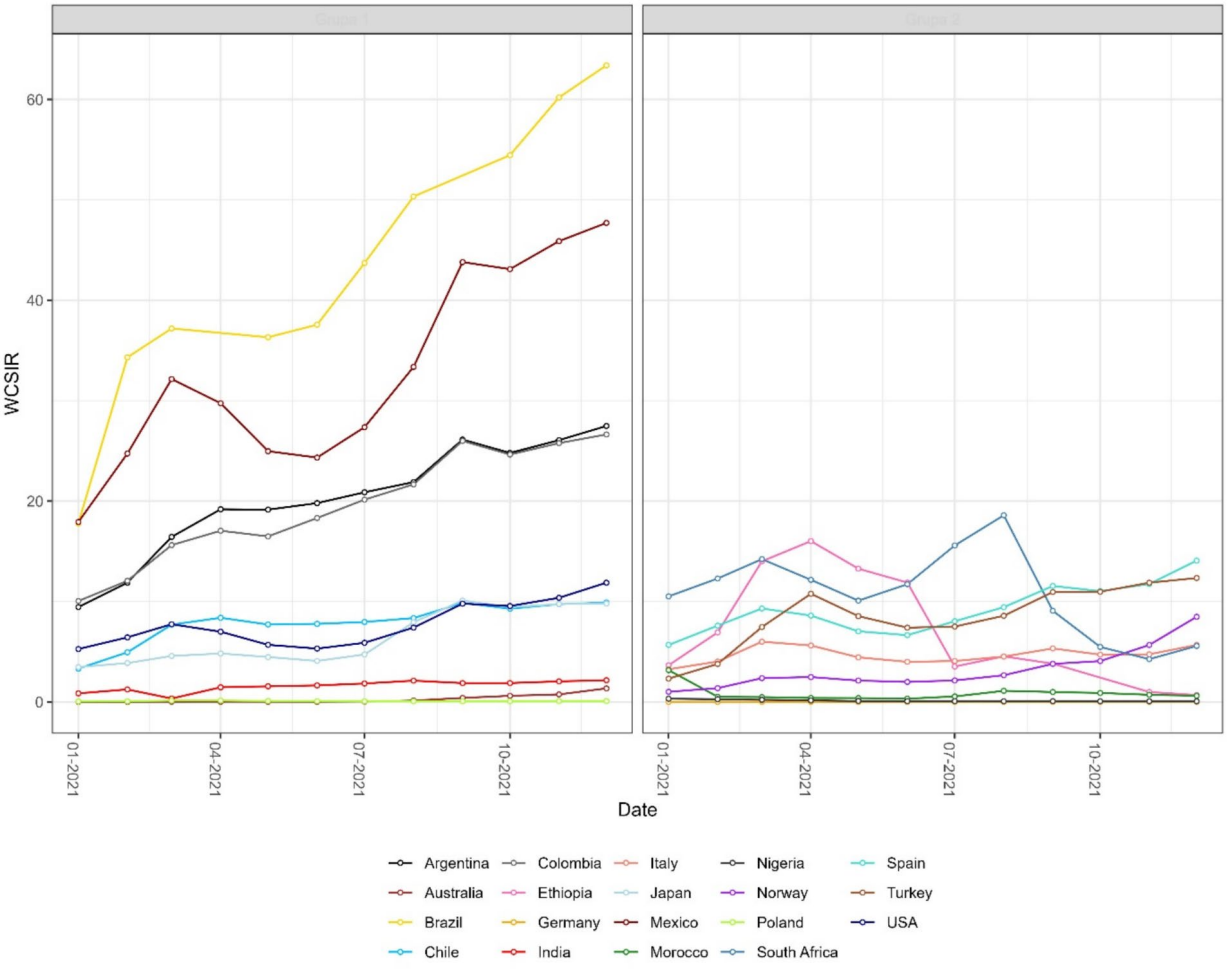
Monthly time series of the weighted cumulative standardized incidence ratio (WCSIR) for selected countries. WCSIR is defined as the cumulative ratio of local to global test positivity rates, capturing long-term differences in viral detectability. Values above 1 indicate that, cumulatively, a country has had a higher test positivity rate than the global average.

**Figure 7:**
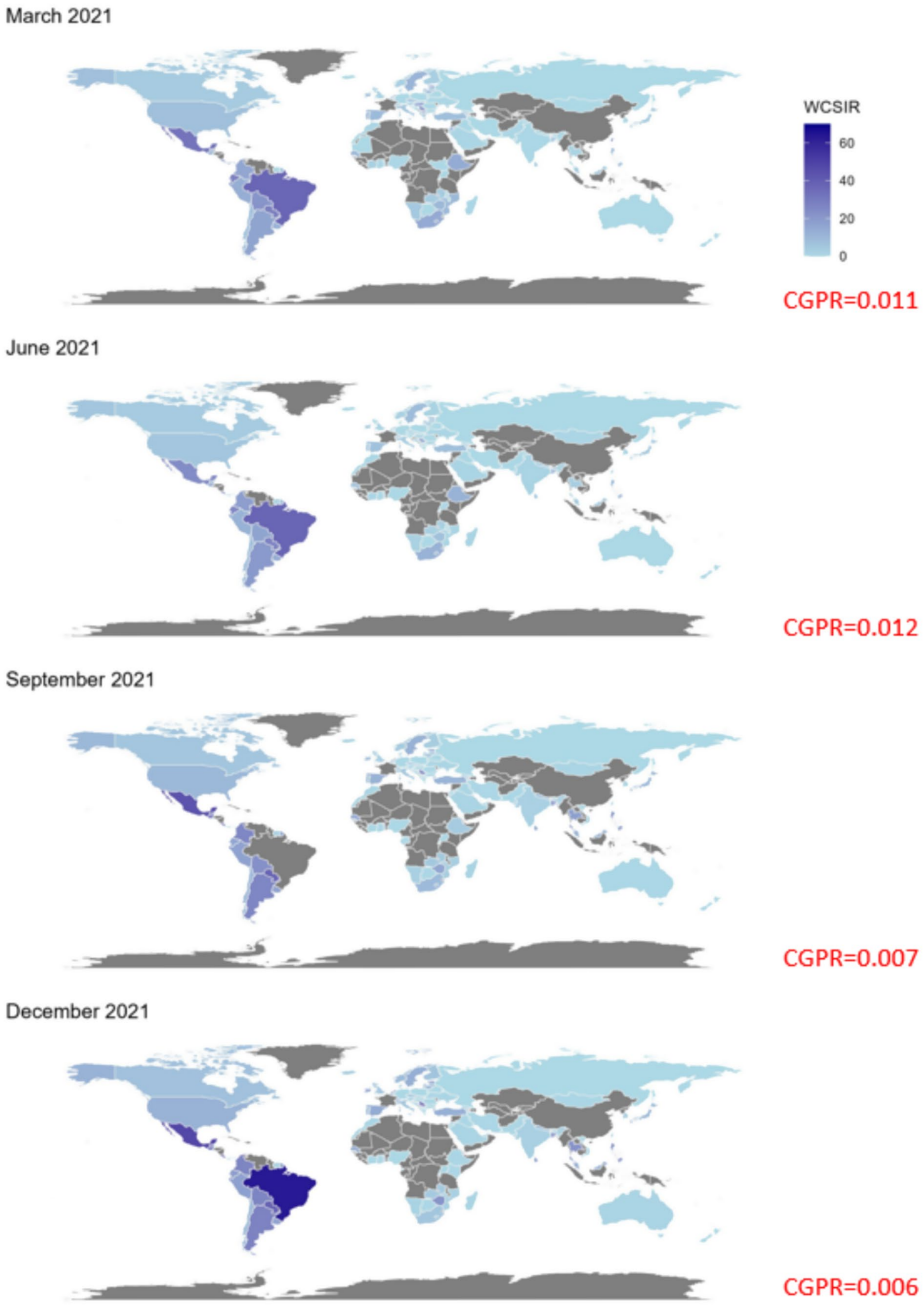
Global maps of the weighted cumulative standardized incidence ratio (WCSIR) for four selected months in 2021: March, June, September, and December. WCSIR is defined as the cumulative ratio of local to global COVID-19 test positivity rates, capturing long-term relative differences in detectability. Higher values (darker shades) indicate countries with persistently higher positivity than the global average, suggesting a sustained combination of under-testing and elevated transmission intensity. Grey shading denotes missing data. The cumulative global positivity rate (CGPR) is reported in red for each month.

**Figure 8:**
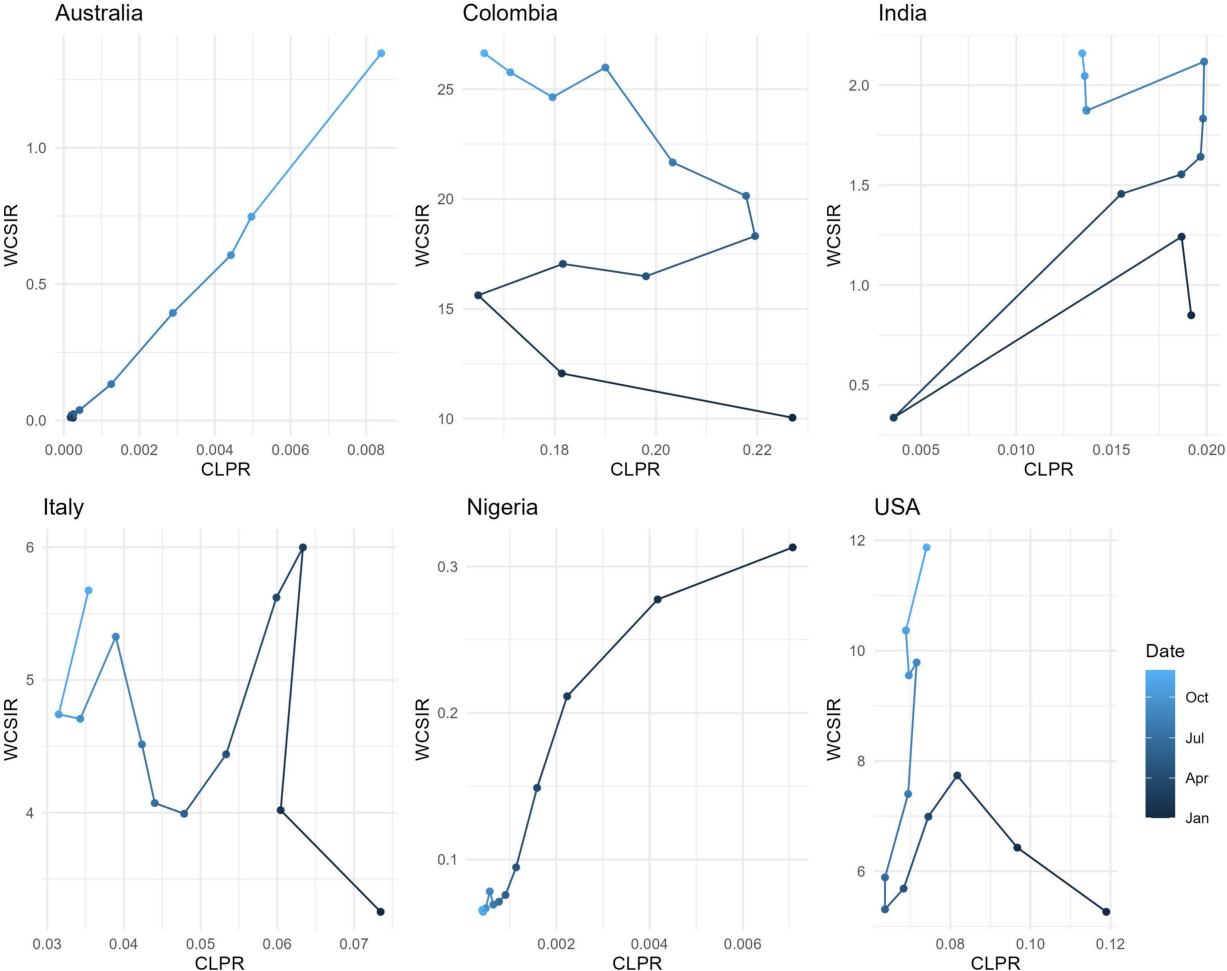
Relationship between the cumulative local positivity rate (CLPR) and the weighted cumulative standardized incidence ratio (WCSIR) for selected countries. Each trajectory represents values from January to December 2021, with shading indicating time progression. While WCSIR often increases with CLPR (e.g., Australia), in some countries WCSIR rises even when CLPR decreases (e.g., Colombia). This reflects changes in the global positivity rate (CGPR), as WCSIR is influenced by both local trends and the evolving global context.

Both instantaneous (Figures 4, 5) and cumulative (Figures 6-8) metrics were mapped. Both versions indicate greater disease burden in South and Middle America reflected by very high WSIR and WCSIR values. For example, from January 2021 to August 2021, the WCSIR value for Argentina was 21.9, for Brazil it was 50.3, for Ecuador it was 12.4, and for Mexico it was 33.4. Correspondingly, there were enormous differences between global (0.94%) and local positivity rates: 20.5% for Argentina, 47.2% for Brazil, 11.7% for Ecuador and 31.3% for Mexico. Surprisingly, COVID-19 burden was not as high for these countries when we do not account for testing coverage (CSIR = 3.28 for Argentina, 2.56 for Brazil, 0.69 for Ecuador, and 0.63 for Mexico). The phase portrait (Figure 8) exhibits trajectories of the relationship between LPR and WCSIR. We note four general patterns: LPR and WCSIR both increasing (Australia); LPR increasing or decreasing, while WCSIR only increases (Colombia); LPR decreasing, but WCSIR decreasing or increasing (Italy); and LPR and WCSIR both decreasing (Nigeria).

### 4.4 Spearman rank correlation analysis

**Figure 9:**
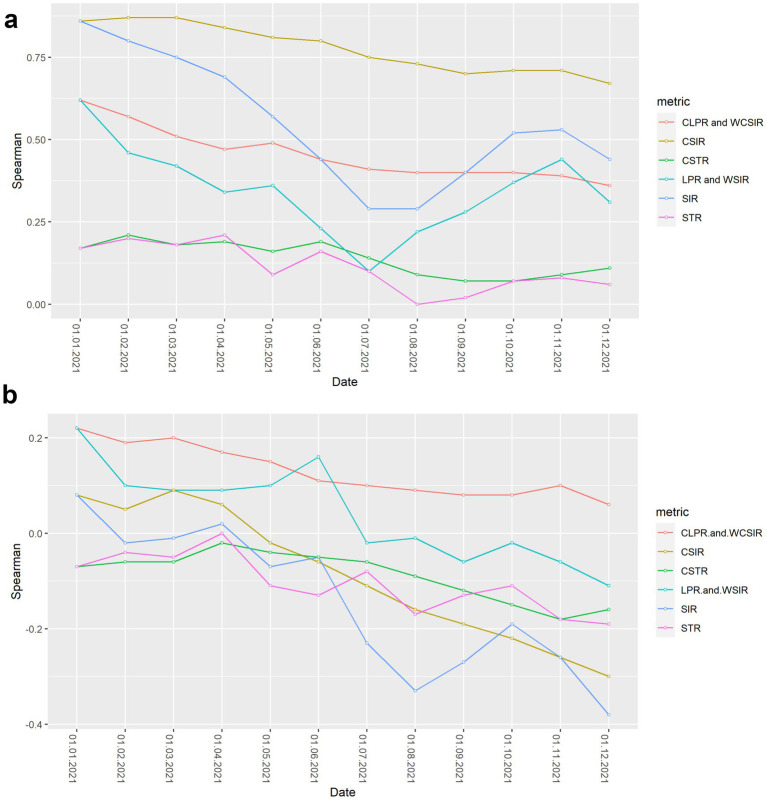
Spearman rank correlation between risk metrics and COVID-19 severity outcomes over time. **(a)** Correlation between risk metrics and cumulative COVID-19 mortality rate. **(b)** Correlation between risk metrics and cumulative case fatality rate.

Results from the Spearman rank correlation analysis for COVID-19 mortality rate and case fatality rate are included in Figures 9a,b, respectively. Full results are in the Supplementary Tables 1, 2.

For the entire study period, all metrics considered were positively correlated with cumulative COVID-19 mortality rate, showing concordance between most risk metrics and mortality burden. Notably, the weakest correlations occurred for metrics that only considered testing coverage (STR and CSTR), suggesting that testing strategies implemented across countries were only weakly associated with COVID-19 mortality.

In general, positive correlations for all metrics with the case fatality rate were weaker than for the mortality rate and became progressively more negative through the study period. The only metrics with a positive correlation with case fatality rate by the end of the study period were observed for the CLPR and WCSIR ( ρ = 0.1–0.2); however, these correlations were not statistically significant (Supplementary Table 2).

## 5 Discussion

### 5.1 Methodology review

According to the World Health Organisation (26, 27) an infodemic is too much information, including false or misleading information, in digital and physical environments during a disease outbreak. It causes confusion and mistrust in health authorities (28, 29). As of February 14, 2023, 132 states signed a cross-regional statement on the COVID-19 infodemic. As discussed, even flawed methodologies from reputable public health institutes contributed the COVID-19 infodemic by using measures that were convenient instead of the best possible available. For example, test positivity was eliminated from a risk classification procedure due to the widespread use of at-home tests (*Test positivity has limited current utility due to the widespread use of point-of-care and at-home tests, and thus was eliminated*.) (1). Challenges with the interpretability of some metrics for lay audiences can also serve as a justification for a metric’s removal [*Metrics that reflect percent change (*e.g.*, in new hospital admissions or new cases) were eliminated due to challenges with interpretability of this metric for lay audiences*] (1).

A significant problem with the CDC approach is that the testing capacity is considered a secondary, qualitative criterion, which suggests it is less important and a lower priority for acceptable measurement. Another problem is that the weights of primary and secondary measures are not given, which raises questions about transparency of the procedure to classify risk in regions. Moreover, with the development of the pandemic, the initial thresholds classifying risk will need continuous modifications as health threats evolve. The Robert Koch Institute adopted a similar strategy by including testing rates into secondary set of criteria labelled as “qualitative and other”, the weights of which were also not provided and reduce the transparency and interpretability of the risk estimates (9).

We note that the testing data was implicitly used by the CDC by calculating the test-to-case ratio defined as the number of tests conducted for each case reported during a fixed period of time (30). We argue, however, that this number is more difficult to interpret than its inverse (positivity rate) because the test-to-case ratio is always greater than one, which does not allow probabilistic interpretation.

The main problem with the approach adopted by the UKSHA can be the material redundancy of criteria in that the positivity rate can be expressed as the ratio of the case rate to the test rate, which, when considered separately, are not epidemiologically meaningful: case rate is sensitive to testing rates and test rate is not capable of assessing the disease burden. Although assigning weights to the criteria could be a solution to the problem, we believe that the simplicity and interpretability of combining the two metrics into one would better serve to inform ministerial decisions. A similar issue can be observed in the Africa CDC COVID-19 dashboard (31), which presents raw counts of cases and tests in parallel, without synthesizing them into an integrated indicator such as test positivity. This structural separation of closely related metrics may unintentionally amplify the impact of testing variability on risk assessments and illustrates that redundancy of indicators is a broader issue affecting surveillance systems beyond high-income countries.

Covid ActNow eliminated the use of test positivity in spring 2022 to assess risk levels due to increasing number of unreported at-home tests (15). The updated criteria include cases (weekly incident cases per 100,000), admissions (weekly COVID-19 admissions per 100,000), and patients (% of beds with patients with COVID-19). However, Covid ActNow acknowledged that test positivity can still be used by “people who need extra caution” (15). We acknowledge that the public health agencies operated under extraordinary time and resource constraints during the pandemic, which likely influenced their methodological choices; while our critique focuses on technical aspects, it does not intend to overlook the complexity of the operational context.

### 5.2 Spatial and statistical insights

The probabilistic standardization yielded striking contrasts between South American countries and the rest of the world that otherwise could remain unnoticed. The mean value of instantaneous SIR for South American countries was 1.88, which is close to the mean of 1.76 across all the countries included. However, the mean value of instantaneous LPR for South American countries was 14.62, which is far greater than the mean value of instantaneous GPR (0.84). Two examples can be given with respect to the observed contrasts: (1) between population-based and probabilistic risk metrics and (2) between relative infection burden and testing coverage. The greatest contrast between population-based and probabilistic risk metrics can be observed for Ecuador. Ecuador’s CSIR was never greater than 1 and its mean WCSIR of 19.5 indicated much higher LPR than GPR. It is worth noting that Ecuador’s testing rates were extremely low (CSTR always below 0.1). An even greater difference between local and global positivity rate can be observed for Brazil (mean WCSIR = 43.5), indicating much higher relative disease burden than testing coverage. In contrast to Ecuador, Brazil achieved above-average CSIR values (mean CSIR = 2.50). Interestingly, these above average relative infection burden values were achieved despite extremely low testing rates (mean CSTR = 0.065). It was widely reported that Brazil did not undertake any substantial COVID-19 testing or control strategies (32, 33).

Given these differences, and assuming that disease spread exhibits spatial autocorrelation, it is likely that the CSIR estimate does not adequately capture the synchronization of epidemics, as it omits spatial heterogeneity in testing practices. The strongest correlations with COVID-19 mortality rates was observed for CSIR and SIR values. This strong correlation with population-based metrics speaks to COVID-19 deaths being largely composed as a subset of confirmed cases. If this is the case, a low position in the CSIR ranking would imply a low position in the mortality rate ranking, a reinforcing feedback loop that results in the underreporting of deaths when low testing coverage is not taken into account.

In contrast, population-based risk metrics did not show the strongest correlations with the case fatality rate. Slightly higher (though statistically non-significant) correlations were observed for CLPR and WCSIR. These exploratory patterns may suggest that probabilistic measures capture complementary aspects of disease severity, but further validation is needed before drawing firm conclusions.

Spearman correlation analysis has already been applied in COVID-19 research (34–36); however, these approaches did not investigate the sensitivity of correlation results on mortality through time. This temporal aspect can generate new hypotheses about the relationship between COVID-19 surveillance and outcomes. For example, one such interpretation of a change from weak positive correlation to negative correlation between SIR and case fatality rate (Figure 9B) could be countries conducting more extensive testing over time, leading to the identification of additional mild or asymptomatic cases. This increased detection might result in a lower overall case fatality rate, as a higher proportion of less severe cases are captured in the data. In this study, we did not disaggregate by specific SARS-CoV-2 variants or epidemic phases, as our goal was to present an aggregated view of risk indicators across countries for the entire year 2021. Future research could explore how these metrics perform across distinct phases of the pandemic, including periods dominated by specific variants or shaped by differing levels of vaccine uptake.

### 5.3 Dual interpretations of the WSIR metric

The Weighted Standardized Incidence Ratio (WSIR) can be conceptualized in two ways. One formulation defines WSIR as the ratio of the Standardized Incidence Ratio (SIR) to the Standardized Testing Ratio (STR), which requires population denominators (see Table 1) and follows the established principles of indirect standardization (18). In this formulation, the global incidence rate—calculated as the proportion of confirmed cases to total population across all regions—is applied to estimate the expected number of cases in a specific region, assuming it experienced the same risk as the global average. The SIR is then calculated as the ratio of observed to expected cases. This approach provides a statistically grounded baseline for evaluating whether a region’s incidence exceeds what would be anticipated under uniform risk distribution.

However, an alternative and operationally simpler formulation defines WSIR directly as the ratio of the local to global test positivity rates (i.e., confirmed cases divided by conducted tests), without relying on population data (4). Despite its simplicity, it preserves the conceptual intent of WSIR—namely, to detect disproportionate case burden relative to testing effort—while increasing practical applicability.

Therefore, WSIR quantifies whether the proportion of positive tests in a given region is higher or lower than what would be expected under a global-average positivity benchmark. In this sense, WSIR functions as an early-stage analytical tool, similar to a probabilistic benchmark or controlled experiment (e.g., a fair coin toss), providing a first-level signal of potentially anomalous patterns in disease detection. More complex structural and epidemiological factors—such as health system disparities, behavioral determinants, or differences in prior immunity—should be incorporated in subsequent, more granular analyses.

### 5.4 Limitations

This study has several limitations. Not all countries reported daily numbers of cases, mortality and tests, and our study was limited to only those countries without missing data. Therefore, the results presented in this study are based on a subset of countries with complete data and may not fully generalize to excluded nations, particularly those with limited data availability. Potential selection bias cannot be ruled out, especially in relation to geographic, economic, or health system characteristics of countries not included in the analysis. Furthermore, countries reporting cumulative data at irregular intervals were also excluded from the analysis. Moreover, the definition of COVID-19 mortality is unspecified and it may vary across countries. For example, some countries may include only deaths with laboratory-confirmed infection, while others may also count suspected or probable cases based on clinical criteria. These discrepancies in classification and reporting practices can lead to substantial differences in recorded mortality figures. As a result, the strength and direction of correlations involving mortality—such as those explored in this study—may be distorted. In particular, inconsistent definitions can introduce systematic biases that either attenuate or exaggerate observed associations, thus reducing the reliability and comparability of the correlation coefficients across countries. Future studies should aim to use more standardized or harmonized mortality datasets, where available, to minimize uncertainties related to inconsistent definitions and improve the comparability of findings across countries. Regarding the Spearman correlation analyses, it is important to note that the Case Fatality Rate (CFR) shares its denominator—confirmed cases—with several metrics used in our framework, such as test positivity (used in WSIR and WCSIR). As a result, correlations between CFR and our probabilistic indicators may partially reflect structural dependencies introduced by testing practices rather than independent epidemiological relationships. These correlations should therefore be interpreted with caution, especially in contexts where causal inference is not the primary goal.

We note that the created dashboard (see the Computer Code Availability section) primarily serves to illustrate the discussed concepts, and it may be premature to consider it a tool ready for use by decision-makers. With regard to the contribution of opaque risk metrics to the infodemic, we believe the study specifically addresses this metric-related aspect, rather than proposing a comprehensive solution to the broader phenomenon. Regarding the adaptive value of public health institutions’ risk assessment frameworks, we note that UKHSA previously employed materially redundant indicators—treating case counts and test positivity as independent criteria (16)—in their international travel risk classification methodology, potentially amplifying testing-related biases. However, in their current domestic surveillance dashboard (37), test positivity is reported as a key indicator. Similarly, the CDC dashboard highlights test positivity as the leading ‘early indicator’ (38), reflecting a shift toward metrics that are more robust to variations in testing intensity and timing. It is worth noting that the ‘global’ reference in our standardization framework does not necessarily refer to the entire world, but can be flexibly applied to any defined population—such as a set of regions within one country or a group of localities under a shared public health policy. In such settings, the assumption of uniform exposure risk may be more plausible and may help isolate epidemiological signals rather than structural or infrastructural differences.

## 6 Conclusion

In this study, we critiqued COVID-19 disease burden classification methods adopted by various influential public health institutes and calculated rankings of COVID-19 disease burden that incorporated testing coverage across countries over time. These rankings are based on a global reference value for test positivity, which serves as a benchmark for assessing relative risk levels across countries. According to our rankings, South American countries in 2021 had a disproportionately high burden of COVID-19 when taking testing into account, a burden that was masked when using population-based metrics only. Population-based metrics showed stronger and statistically significant correlations with COVID-19 mortality rates, while probabilistic metrics exhibited weak, non-significant correlations with case fatality rates. Further analysis between these metrics and other COVID-19-related outcomes should assess the predictive value of probabilistic metrics.

### Computer code availability

Name of code: COVID License: GNU General Public License v3.0. Developer: Elżbieta Węglińska. Contact address: AGH University of Science and Technology, Krakow, Poland. E-mail: weglinska@agh.edu.pl. Year first available: 2023. Software required: RStudio. Program language: R Program size: 16KB. How to access the source code: Available at: https://github.com/weglinska/COVID. The dashboard is available at: https://agh-ust.maps.arcgis.com/apps/dashboards/e5b031aa401d47f9a5ffac0be8f3662b#locale=en.

## Data Availability

Data used in this study were accessed in Guidotti (17) (doi: 10.1038/s41597-022-01245-1). Processed data are available at: https://github.com/weglinska/COVID.
