# Supplementary material for: COVID-19 global risk evaluation: rankings, reducing surveillance bias, and infodemic
Source: Front Public Health. 2025 Aug 4;13:1589461. doi: 10.3389/fpubh.2025.1589461 (PMC12358430; doi:10.3389/fpubh.2025.1589461)
Supplement: Supplementary file 1 [file Supplementary_file_1.docx]

**Supplementary information**

**Supplementary Table S1** Spearman rank correlation - p-values corresponding to the test of statistical significance of the Spearman rank correlation coefficient between various risk metrics and an outcome variable defined as cumulative deaths/population.

| **Date** | **CLPR** | **WCSIR** | **LPR** | **WSIR** | **CSIR** | **SIR** | **CSTR** | **STR** |
| --- | --- | --- | --- | --- | --- | --- | --- | --- |
| **01.01.2021** | <0,000001 | <0,000001 | <0,000001 | <0,000001 | <0,000001 | <0,000001 | 0,08 | 0,08 |
| **01.02.2021** | <0,000001 | <0,000001 | 0,000001 | 0,000001 | <0,000001 | <0,000001 | 0,04 | 0,05 |
| **01.03.2021** | <0,000001 | <0,000001 | 0,000015 | 0,000015 | <0,000001 | <0,000001 | 0,08 | 0,08 |
| **01.04.2021** | 0,000001 | 0,000001 | 0,00048 | 0,00048 | <0,000001 | <0,000001 | 0,06 | 0,03 |
| **01.05.2021** | <0,000001 | <0,000001 | 0,0002 | 0,0002 | <0,000001 | <0,000001 | 0,11 | 0,36 |
| **01.06.2021** | 0,000003 | 0,000003 | 0,021 | 0,021 | <0,000001 | 0,000005 | 0,06 | 0,12 |
| **01.07.2021** | 0,000022 | 0,000022 | 0,30 | 0,30 | <0,000001 | 0,0029 | 0,17 | 0,34 |
| **01.08.2021** | 0,000028 | 0,000028 | 0,026 | 0,026 | <0,000001 | 0,0033 | 0,36 | 0,96 |
| **01.09.2021** | 0,000041 | 0,000041 | 0,0048 | 0,0048 | <0,000001 | 0,000027 | 0,48 | 0,87 |
| **01.10.2021** | 0,000039 | 0,000039 | 0,00013 | 0,00013 | <0,000001 | <0,000001 | 0,46 | 0,50 |
| **01.11.2021** | 0,000048 | 0,000048 | 0,000005 | 0,000005 | <0,000001 | <0,000001 | 0,36 | 0,41 |
| **01.12.2021** | 0,00022 | 0,00022 | 0,0017 | 0,0017 | <0,000001 | 0,000004 | 0,30 | 0,52 |

**Supplementary Table S2** Spearman rank correlation - p-values corresponding to test of statistical significance of the Spearman rank correlation coefficient between various risk metrics and an outcome variable defined as cumulative deaths/cumulative confirmed cases.

| **Date** | **CLPR** | **WCSIR** | **LPR** | **WSIR** | **CSIR** | **SIR** | **CSTR** | **STR** |
| --- | --- | --- | --- | --- | --- | --- | --- | --- |
| **01.01.2021** | 0,03 | 0,03 | 0,03 | 0,03 | 0,45 | 0,45 | 0,48 | 0,48 |
| **01.02.2021** | 0,06 | 0,06 | 0,32 | 0,32 | 0,62 | 0,88 | 0,52 | 0,70 |
| **01.03.2021** | 0,04 | 0,04 | 0,36 | 0,36 | 0,40 | 0,96 | 0,58 | 0,65 |
| **01.04.2021** | 0,09 | 0,09 | 0,36 | 0,36 | 0,55 | 0,81 | 0,84 | 1,00 |
| **01.05.2021** | 0,13 | 0,13 | 0,30 | 0,30 | 0,83 | 0,49 | 0,67 | 0,27 |
| **01.06.2021** | 0,25 | 0,25 | 0,12 | 0,12 | 0,56 | 0,65 | 0,60 | 0,21 |
| **01.07.2021** | 0,34 | 0,34 | 0,87 | 0,87 | 0,27 | 0,02 | 0,52 | 0,42 |
| **01.08.2021** | 0,38 | 0,38 | 0,95 | 0,95 | 0,11 | 0,00 | 0,36 | 0,09 |
| **01.09.2021** | 0,45 | 0,45 | 0,54 | 0,54 | 0,05 | 0,01 | 0,24 | 0,18 |
| **01.10.2021** | 0,41 | 0,41 | 0,87 | 0,87 | 0,03 | 0,06 | 0,13 | 0,27 |
| **01.11.2021** | 0,34 | 0,34 | 0,58 | 0,58 | 0,01 | 0,01 | 0,07 | 0,06 |
| **01.12.2021** | 0,53 | 0,53 | 0,29 | 0,29 | 0,00 | 0,00 | 0,11 | 0,05 |
